# Proactive Assessment of Obesity Risk during Infancy (ProAsk): a qualitative study of parents’ and professionals’ perspectives on an mHealth intervention

**DOI:** 10.1186/s12889-019-6616-5

**Published:** 2019-03-12

**Authors:** Jennie Rose, Cris Glazebrook, Heather Wharrad, A. Niroshan Siriwardena, Judy Anne Swift, Dilip Nathan, Stephen Franklin Weng, Pippa Atkinson, Joanne Ablewhite, Fiona McMaster, Vicki Watson, Sarah Anne Redsell

**Affiliations:** 1 Faculty of Health, Education, Medicine and Social Care, Anglia Ruskin University, East Road Campus, Cambridge, England; 2 Institute of Mental Health, University of Nottingham Innovation Park, Nottingham, England; 3 School of Health Sciences, University of Nottingham, Nottingham, England; 4 Community and Health Research Unit, School of Health and Social Care, University of Lincoln, Lincoln, England; 5 Division of Nutritional Sciences, School of Biosciences, University of Nottingham, Nottingham, England; 6 Nottingham University Hospitals Trust, Nottingham, England; 7 Division of Primary Care, University of Nottingham, Nottingham, England; 8 Nottingham City Care Partnership, Nottingham, England; 9 Faculty of Medical Sciences, Anglia Ruskin University, Cambridge, England

**Keywords:** Childhood obesity, Infant, Prediction, Prevention, Parents, Health visitor, Risk communication, mHealth

## Abstract

**Background:**

Prevention of childhood obesity is a public health priority. Interventions that establish healthy growth trajectories early in life promise lifelong benefits to health and wellbeing. Proactive Assessment of Obesity Risk during Infancy (ProAsk) is a novel mHealth intervention designed to enable health professionals to assess an infant’s risk of future overweight and motivate parental behaviour change to prevent childhood overweight and obesity. The aim of this study was to explore parents’ and health professionals’ experiences of the overweight risk communication and behaviour change aspects of this mHealth intervention.

**Methods:**

The study was conducted in four economically deprived localities in the UK. Parents (*N* = 66) were recruited to the ProAsk feasibility study when their infant was 6–8 weeks old. Twenty two health visitors (HVs) used a hand-held tablet device to deliver ProAsk to parents when their infants were 3 months old. Parents (*N* = 12) and HVs (*N* = 15) were interviewed when infants in the study were 6 months old. Interview data were transcribed and analysed thematically using an inductive, interpretative approach.

**Results:**

Four key themes were identified across both parent and health visitor data: Engaging and empowering with digital technology; Unfamiliar technology presents challenges and opportunity; Trust in the risk score; Resistance to targeting. Most participants found the interactivity and visual presentation of information on ProAsk engaging. Health visitors who were unfamiliar with mobile technology drew support from parents who were more confident using tablet devices. There was evidence of resistance to targeting infants at greatest risk of future overweight and obesity, and both parents and health visitors drew on a number of reasons why a higher than average overweight risk score might not apply to a particular infant.

**Conclusions:**

An mHealth intervention actively engaged parents, enabling them to take ownership of the process of seeking strategies to reduce infant risk of overweight. However, cognitive and motivational biases that prevent effective overweight risk communication are barriers to targeting an intervention at those infants most at risk.

**Trial registration:**

NCT02314494. Date registered 11th December 2014.

**Electronic supplementary material:**

The online version of this article (10.1186/s12889-019-6616-5) contains supplementary material, which is available to authorized users.

## Background

Obesity during childhood has serious adverse effects on the physical health and psychosocial well-being of children [1–3] and is associated with poorer health later in life [4]. Worldwide, over 41 million children under the age of five were overweight in 2016 [5] and addressing the upward trend in childhood obesity is a public health priority. The WHO Commission on Ending Childhood Obesity identified early life as one of the critical periods for obesity prevention [6] and postnatal interventions that target infant diet and parental responsiveness show promise [7]. However, few interventions have demonstrated beneficial effects on infant growth patterns and there have been calls for novel approaches to be developed [8].

Proactive Assessment of Obesity Risk during Infancy (ProAsk) is a novel mHealth intervention developed to identify infant overweight risk and prevent childhood obesity. Drawing on the extended health belief model of behaviour change [9] it seeks to increase parents’ understanding of their infant’s risk of child overweight and promote self-efficacy for behaviour change. Incorporating a validated risk prediction algorithm [10, 11] ProAsk supports health professionals to quantify and communicate an infant’s overweight risk status. An interactive therapeutic wheel, based on a systematic review of interventions to prevent childhood overweight and obesity, facilitates a motivational discussion about behaviour change. ProAsk thus enables health professionals to identify infants at increased risk of future overweight, and to target prevention to vulnerable families.

Personalised risk communication is intended to improve awareness of health risks and promote risk-reducing behaviour [12]. Recent advances in interactive digital technology have shown promise as resources for delivering personalised health information to improve health outcomes [13, 14]. Dietitians recognise the potential of digital resources to support communication with children and parents around the sensitive topic of child obesity [15], but there is a paucity of research into the use of mHealth interventions that support communication in child-focussed clinical settings [10, 16]. Evidence from other arenas is encouraging. Handheld tablet devices have been reported to raise engagement in learning in non-health settings [17, 18]. Studies in adult-focussed health settings suggest that interactive digital technology has potential to improve clinicians’ communication [19], facilitate the exchange of complex and sensitive discussion between health professionals and patients to improve care [20–23] improve patients’ knowledge and encourage protective behaviour change [24].

This study aimed to explore parent and health professional experiences of using digital technology for Proactive Assessment of Obesity Risk during Infancy (ProAsk). It was conducted in the context of a feasibility study which examined the feasibility and acceptability of undertaking a randomised controlled trial of ProAsk with UK health visitors (HVs) and parents [25]. The current study sought to understand the processes by which ProAsk seeks to effect change by exploring parents’ and health visitors’ perspectives of both the overweight risk communication and the motivational behaviour change element of this interactive digital intervention.

## Methods

### Design

This was a qualitative interview study with HVs and parents. All participants had used ProAsk, an infant overweight risk assessment and behaviour change intervention. Our methodological approach was informed by a critical realist perspective. This philosophical stance assumes that reality is socially constructed through language, but that these constructions are shaped by the material world [26]. We used the checklist for the consolidated criteria for reporting qualitative studies (COREQ) [27], and the completed checklist is available in Additional file 1.

### Setting, recruitment and participants

The study was conducted in 2015/16 in four study sites situated in the East of England. Two of these sites were urban, and two were rural. In order to maintain the anonymity of participants specific study sites are not named. The sites were chosen to be ethnically diverse and with a higher proportion of children living in income deprived families than is the average for England, because overweight and obesity are more prevalent among these groups [28, 29].

The recruitment and procedure for the feasibility study are detailed in Redsell et al. [25]. In brief, HVs identified eligible parents at a routine 6–8 week infant check. Those that gave permission for their details to be passed onto the research team were visited by a researcher who took their informed written consent to take part in the feasibility study and to take part in interviews at the end of the study. Sixty-six parents and 22 HVs took part in the feasibility study. The feasibility study found limited evidence to support the feasibility of implementing ProAsk due to problems with study recruitment and protocol adherence [25].

All of the HVs who had given their informed written consent to take part in the ProAsk feasibility study and who were still in post at the time of the interviews were emailed or telephoned and invited to take part in the interviews. Fifteen agreed to take part. The seven that did not take part had left post, were on leave, or did not have time to take part in the interviews. All health visitors and parents who took part in interviews verbally reconfirmed their consent to take part in the interviews prior to the interviews taking place.

Consistent with the exploratory nature of this qualitative study, we used purposive sampling to identify a maximum variation sample of the parents who had received ProAsk. Twelve parents were telephoned when their infants were six months old and invited to take part in the interviews; all agreed to take part. The sample included parents from all four study sites. Six of these parents had infants who were identified as being at above average risk of future overweight, and the remaining six had received an average risk of overweight result for their infant.

### The mHealth intervention

HVs used a hand-held tablet device to deliver ProAsk to parents when their infants were 3 months old. HVs entered the IROC algorithm [10] items (baby birth weight, current weight and length, maternal and paternal weight, maternal smoking status during pregnancy) into ProAsk which then calculated the infant’s overweight risk. The infant’s risk status was displayed on the tablet screen as either “Your baby’s risk of being above a healthy weight is the same as other babies” (population risk) or “Your baby’s risk of being above a healthy weight is more than other babies” (above population risk). For parents whose infants were above population risk of overweight, the program prompted HVs to conduct a short motivational interview supported by an interactive graphic which promoted evidence-based behaviour change strategies [7] in four areas: active play; milk and solid foods; sleeping and soothing; and infant feeding cues. HVs received motivational interviewing (MI) [30] refresher training, and were encouraged to use these techniques to build parental self-efficacy for behaviour change.

### Data collection

At the start of the ProAsk feasibility study (infant age 2 months) demographic details and ethnicity were collected via a self-report questionnaire completed by parents. At the end of the feasibility study two researchers (JR/JA) conducted semi-structured interviews with parents and HVs. The research team developed the interview guides (Additional file 2: Table S1 and S2) to cover the areas of focus of a feasibility study [31]. Data concerning feasibility of study methods are reported elsewhere [25]. Interviews with parents were conducted over the telephone. Parents were located in their own homes, researchers were in a quiet office. Interviews with HVs were conducted face to face in a quiet room in the clinic/practice where they worked, or over the telephone in the same setting. Interviews lasted 20–90 min, and were audio recorded with a digital recording device. Interview recordings were transcribed verbatim, anonymized and transcripts imported into QSR NVivo 10 software© for data management.

### Data analysis

We analysed the data using thematic analysis, a method for identifying and interpreting patterns across qualitative data that is suited to investigating under-researched topics [32]. Codes and themes were developed using an inductive, interpretative approach [33, 34] across both parent and HV datasets. The aim was to gain an understanding of the participants’ perceptions of infant overweight risk assessment and intervention with ProAsk rather than to determine participants’ answers to particular question. Consistent with our relativist epistemology, the concepts of saturation and member checking were not applied. Five researchers generated preliminary codes inductively through in depth discussion of one parent and one HV interview.

Both the semantic content and more latent meanings in the data (e.g. assumptions and ideas implicit in explicit responses to questions) were coded. Two researchers (JR, SR) then independently coded a further three parent and three HV transcripts. The codes thus generated were organised in related clusters and explored for linked and explanatory themes which were further adjusted following discussion with the research team. A coding book with codes, definitions and examples was developed according to the method of Boyatzis [33] and one researcher (JR) reanalysed the entire dataset using the agreed codes.

## Results

Fifteen HVs and 12 parents were interviewed. All HVs were qualified, with between one year and 36 years health visiting experience. The HVs were predominantly white British and female. Parent and infant participant characteristics are summarised in Table 1.

**Table 1:** Demographic and participant characteristics of the parents (and their infants) who took part in the interviews (*N* = 12) and those who took part in the feasibility study (*N* = 53)

	Interview sample		Feasibility study sample	
Parent participant characteristics	N/Mean	%/SD	N/Mean	%/SD
Income Deprivation Affecting Children				
Index (IDACI), 2015				
Quintile 1 (most deprived)	1	8%	8	15%
Quintile 2	4	33%	10	19%
Quintile 3	3	25%	22	42%
Quintile 4	2	17%	10	19%
Quintile 5 (least deprived)	2	17%	3	6%
Highest education level				
GCSE	3	25%	20	38%
A Levels	0	0%	9	7%
Degree	9	75%	22	41%
Smoking in pregnancy				
No	11	92%	52	98.1%
Yes	1	8%	1	1.9%
Parental BMI and prevalence of overweight				
Mean pre-pregnancy maternal BMI (kg/m^2^)	27.3	23.7	25.9	7.9
Number of mothers pre-pregnancy BMI ≥ 25.00 (overweight)	8	67%	22^1^	43%
Mean Paternal BMI (kg/m^2^)	26.1^1^	4.9	28.0^2^	4.8
Number of fathers above BMI ≥ 25.00 (overweight) (2 missing values)	4	40%	26^2^	68%
Feeding choice at 2 months				
Exclusive breastfeeding	7	58	22	41.5
Mixed formula and breast	3	25	6	11.3
Formula only	2	17	25	47.2
Infant characteristics	N/Mean	%/SD	N/Mean	%/SD
Infant’s gender				
Boy	7	58%	27	51%
Girl	5	42%	26	49%
Ethnicity of infant				
White British	10	83%	47	89%
Non-White British/Mixed/Other	2	17%	6	11%
Infant weight and overweight risk				
Mean Birth weight (kg)	3.7	0.47	3.5	0.5
Number of infants with ProAsk Risk Score above population risk	6	50%	21	40%
Number of infants with ProAsk Risk Score at population risk or below	6	50%	32	60%

^1^2 missing values

^2^15 missing values

Categorical variables are numbers and proportions; continuous variables are means and standard deviations

As the parents were purposively sampled to obtain a balance with regard to infant risk of future overweight, the sample would not be expected to be representative of the overall feasibility study sample. However, Table 1 shows that, for most of the core domains in the sampling frame, parents from the different groups recruited to the feasibility study were represented in the interview sample. Thus, parents interviewed included individuals from every quintile of deprivation, feeding choice, smoking status and ethnicity.

Thematic analysis of the interview transcripts identified four key themes: Engaging and empowering with digital technology; Unfamiliar technology presents challenge and opportunity; Trust in the risk score; Resistance to targeting. These themes were evident across the dataset, for parents and HVs, and for parents with infants who differed with respect to their infant’s risk of future overweight. The four themes are presented sequentially with illustrative verbatim quotes. In order to minimise the risk of the identification of participants, the overweight risk status of a parent’s infant is not shown.

### Theme 1: Engaging and empowering with digital technology

Most participants suggested the information presented on ProAsk was visually engaging. HVs found that parents were keen to explore the programme. Part of the perceived usefulness of ProAsk was attributed to the information being presented in an accessible, easy-to-follow manner. In addition, HVs and parents recognised that ProAsk facilitated conversations about what could be a sensitive topic.

*“I thought the information that was in there was really nice and visual actually, because sometimes you can hear a lot of information and it’s sort of difficult to absorb it and there was quite a lot of it, it was nice to have something in front of you as well, as you were having that discussion. It was a sort of a visual prompt that you could refer back to.”* (Parent 8)

*“It [ProAsk] led into that conversation without it being too awkward because you’re already talking about it. It flowed a bit better, if that makes sense. It sort of opened the door a little bit for discussion of obesity and the impact later on and through childhood and into adulthood.” (HV 35)*

The informational content of ProAsk was valued by HVs and parents alike. HVs perceived that parents were keen to explore the programme, accessing the different sections of the ‘therapeutic wheel’ graphic to bring up information about feeding, physical activity, and sleep or soothing. For example, one health visitor (HV13) commented: ‘I felt that the tool was quite simple, brightly coloured, parents liked it, very visual and they were able to choose what was important to them.’

HVs suggested that, rather than imparting information to parents in a didactic manner, digital technology empowered parents to take control of the interaction. The interactivity of the programme was perceived as complementing the parent-led motivational techniques used by HVs to promote behaviour change.

*“I think the tool was very good. I liked that. I think parents liked it. It’s quite simple, very visual. And that really throws the emphasis back onto the parent to say ‘What’s important to you then? What area shall we cover here?’ and that’s all that motivational stuff that leads on.”* (HV 46)

HVs also felt that digital technology could enhance parents’ understanding by enabling them to drive their own learning. They were able to explore the programme at their own pace, following aspects that were pertinent to them, then use this new knowledge to engage in personalised discussions with the HV. Parents echoed the importance of the programme’s interactivity.

*“Reading the information it sits in your mind giving, well making you more aware and more conscious about what you’re feeding your baby. Cos it was interactive it’s made me aware to make better or different choices.*” (Parent 56)

*“And again, them being in control they can read it at their own, rather than actually somebody holding the tablet and driving for them, they can go at their own pace, digest it and then ask the questions really without feeling like someone’s hovering over them*” (HV 43)

### Theme 2: Unfamiliar technology presents challenge and opportunity

HVs were not used to using digital technology in consultations, and initially some found it a struggle. They wanted to look professional and feel confident using the tablet, but inexperience with the technology made this a challenge. However, most reported that with practice and support from their team they grew more proficient. Parents were aware of HVs’ inexperience with the tablet and the effort required to deliver ProAsk. They recalled attempts to resolve technical issues, and some felt that additional training might be beneficial.

*“The first one wasn’t that hot but it got better, we all fiddled around in the office a bit, had a bit of a laugh actually, how do we do this again? I mean it’s very simplistic….It’s just being in the clinical setting with mum, and really praying it’s going to work, not wanting to look unprofessional*.” (HV 22)

*“Because I remember that when she did come with the with the tool, she hadn’t actually used it and she was trying to find out what the password was and to get into the tool in the first place, so I think maybe some guidance around how to actually do that, might have been a benefit.”* (Parent 8)

Whilst the HVs were not always comfortable using tablet devices during the study, most parents were familiar with and confident using digital technology. Both parents and HVs reported that parents offered and provided technical support. The resulting change in the power relations between HVs and parents was acknowledged and embraced by some, but not all, HVs.

*“What we did, because she was not really quite as good at technology as I was, we sort of helped each other out. She started off to ask questions and sort of fill it in, and then she went ‘Oh I don’t know exactly where to go from here’ and I ended up sitting next to her, more or less doing it myself.”* (Parent 16)

*“We held it together, looking at it together and when there was a difficulty in getting some information in, they had a look at it as well, and that helped. So it was fine*.” (HV 35)

*“You’re embarrassed because they’re showing you how to do it…so I actually then gave them the tool and they really liked that, going through it, reading it on their own without you holding it and kind of pushing the buttons, letting them read it on their own*.” (HV 43)

### Theme 3: Trust in the risk score

This theme concerns HVs’ and parents’ response to the screening tool and how they construed the risk score as being flawed or unreliable, particularly when talking about an above population risk result. Some HVs expressed mistrust in the risk score, suggesting that the presence or absence of certain factors overrode a higher than population risk score. Professional or educated mothers were seen as being protected from overweight risk, even if ProAsk indicated that the infant was at increased risk of overweight. Similarly, some parents suggested that breastfed infants could not be at risk of overweight or obesity because they believed it was not possible to overfeed a breastfed baby.

Interviewer (talking about an infant above population risk of future overweight): “*Did you agree with the result?*” Health visitor: “*No personally I didn’t, knowing my mum, and she has an older child, she is well educated.”* (HV 7)

*“I think because he was breastfed, and I was sort of under the impression that he’d be highly unlikely to have, at baby age anyway, you can’t really overfeed him, a breastfed baby.”* (Parent 22)

The issue of trust in the risk score was also evident in a tendency among the HVs to disregard or discount an above population risk result. For example, although they had been informed that incomplete data for parents’ weights and heights would deliver a potential under-estimate of risk, some HVs attributed above population risk results to incomplete anthropometric data.

*“Yeah there was a risk, but we feel that was due to dad’s weight, cos we weren’t sure of dad’s weight.”* (HV 7)

One parent expressed reservations about the accuracy of the overweight risk assessment, saying that she had expected it to require more detailed information.

*“We weren’t actually putting much in terms of information into the tool itself. I think the only thing that it asked for at that time was, I think it was just baby’s weights and my weight again… there was literally only a couple of very, very minor questions and I was expecting a lot more sort of in depth questions.*” (Parent 8)

### Theme 4: Resistance to targeting

Although HVs recognised that ProAsk offered an opportunity for positive preventative work with infants identified as being at above population risk of overweight, they were anxious about the impact of this on their relationship with the parents. They felt that telling a parent their child had a higher risk of being overweight could be perceived as judgmental and pejorative.

*“Probably I was quite scared to, probably I don’t want to upset that relationship with the parents, probably I didn’t want to tell them their child was going to be obese. And we didn’t really – I’d say that I did a universal visit for all of them, rather than focusing on the percentage at the end of it.*”(HV 46)

Some parents reported that they were not clear what the result was, or what it meant for them and their baby. These parents expressed a desire to receive clear feedback about their infant’s risk score.

*“I answered the questions, but there wasn’t really any direct feedback given to me. … it would be good to make sure that the health visitors do give that feedback in the future.”* (Parent 21)

Parental responses to the feedback of infant overweight risk varied. An average overweight risk score prompted feelings of relief and pleasure, and an acknowledgement that they may have felt differently if their baby had been identified as at above population risk. A few parents had understood a population risk result to mean no risk. One parent who had received a message that their infant was at above population risk of overweight had found it upsetting. Despite this, no parents reported a negative effect on their confidence as a parent, whereas some who had received an average risk of overweight message reported a positive response to the risk feedback.

*“It felt good. I thought well there’s clearly something that I’ve answered that I’ve done to prevent that.”* (Parent 16)

*No, I’m sure that she said that he isn’t, there isn’t any significant risk for him and so there’s nothing else for us to worry about, that’s it.* (Parent 47)

Interviewer: *How did you feel about the feedback of your baby’s risk?* Parent: *I found a bit distressing to hear though if I’m honest.* (Parent 61)

Targeting the intervention to those infants identified at higher population risk was perceived by some HVs to be at odds with their commitment to universal health promotion. Amid competing priorities for staff and time, other HVs suggested it was beyond their scope to offer on-going targeted support to families with infants at risk of overweight; in contrast, some parents who had received an above population risk score expressed a desire for continued support and monitoring.

*“Well I use the therapeutic wheel at every visit, whether it said at risk or not at risk because my sort of motto is not to miss an opportunity... I know when I did the actual wheel, if you like, you said to discuss one topic, we ended up discussing them all.”* (HV 22)

*“If this was, I suppose, to be done again, to actually have somebody in the field of obesity to support families. Probably outside of our health visiting team, if that makes sense. So that you can do intensive work. Unfortunately we don’t have the capacity to do that because our priority is child protection and children in need and more vulnerable families.*”(HV 35)

*“Perhaps there should be, right, because he’s got a higher risk he could be weighed more in the future.”* (Parent 61)

## Discussion

The primary aim of this study was to gain insight into user experiences of ProAsk, a novel mHealth intervention designed to prevent childhood overweight and obesity during infancy. This intervention supports HVs when communicating personalised risk information to parents of infants, and prompts constructive discussion of strategies that parents are motivated to try in order to reduce their child’s risk of overweight. This is the first study to explore health service users’ and health professionals’ views of an intervention to prevent overweight and obesity during infancy during a routine home visit.

This study highlighted the importance of good design principles in developing mHealth interventions. Both parents and HVs valued design features, including the visual appeal and interactivity of ProAsk. Interactivity and visual attractiveness are both attributes that have previously been reported as valuable in communicating health messages [21]. Perceived usefulness and ease of use are key components of technology acceptance in healthcare [35] and were found to be important to participants in this study too. Interactivity of internet-based interventions has been reported to foster a sense of empowerment, for example, with respect to pain management for patients with chronic back pain [36] and nurses caring for children who self-harm [22, 35]. This engagement and sense of empowerment to engender behaviour change warrants further investigation.

ProAsk was not intended to be used directly by parents, but HVs who were unfamiliar with mobile technology drew support from parents who were more confident using tablet devices. This may have resulted in a shift in the power relations between HVs and parents, and coupled with the interactive nature of the programme, which parents welcomed, appears to have stimulated active engagement with the intervention element of ProAsk by parents. Handheld tablet devices have previously been reported to raise engagement in learning for university students [18, 19]. In health care, others have reported the challenge for healthcare workers to use and access technology [37], and the sense of empowerment that mobile technology can bring to patients [35], but to our knowledge this is the first research to suggest a potential association between the two.

A strength of this study was that it investigated both professional and parent views, with the analysis conducted across both parent and health visitor datasets. This led to the unexpected finding that HVs’ relative inexperience with digital technology had benefits for parents, because it enabled parents to take hold of the device and explore it themselves. Whilst this finding indicates a need for health visitors to have more time in training to learn to use the new technology, the positive response of both parents and health visitors to this development suggests that further development of ProAsk should capitalise on the motivational benefits of empowering parents to use the device themselves, perhaps by providing the parents with independent access via a mobile app.

Personalised risk communication is theorised to lead to greater acceptance of the message regarding risk [38]. However, in this study both HVs and parents had difficulty trusting personalised infant overweight risk scores for infants at an above average risk of overweight. Perception of personal risk can be influenced by prior awareness of risk, understanding of the risk and how it is presented [38]. It is also prone to systematic biases [39, 40]. Previous studies of risk communication to adults in a clinical setting have found that systematic biases can influence healthcare practitioners’ communication and patients’ perception of personalised risk feedback [41]. In this study parents and HVs drew on a number of reasons why a risk score might not apply to a particular child, providing evidence of confirmation bias, where judgements about the validity of information are influenced by how that information fits with existing beliefs [40]. HVs rejected above population risk estimates for parents who they believed to be well educated, a finding that is consistent with the representativeness heuristic, where risk judgements are biased by existing stereotypes [40].

The expressions of relief and pleasure by parents receiving the ‘not above average risk of overweight’ feedback for their infant indicates that parents may be susceptible to false reassurance. This unintentional consequence of lower risk feedback has been documented in studies of adult cancer screening [41] but not in obesity risk screening for adults [42].If ProAsk is to be effective in practice it will be important to address these unconscious biases that present barriers to parental understanding of their infant’s overweight risk score.

Delivering and receiving personalised infant overweight risk was emotive for HVs and parents alike. Some HVs were uncomfortable about conveying the infants’ risk scores to parents, and some parents remained unaware of their infant’s risk status after the consultation. The complex and emotive nature of obesity makes raising infant overweight risk a particular challenge. For the HVs, telling parents that their infant was at a higher risk of being an overweight child risked upsetting them. Glossing over above-population risk results may have helped HVs minimise the threat such a judgement could pose to their relationship with parents. In a similar way, UK GPs have previously been found to prioritise their relationships with parents above intervening to improve infant feeding practices [43].

Parents from the UK [44] and US [45] have previously been found to hold themselves responsible for their child’s obesity. Receiving personalised feedback that their child is likely to become overweight may therefore provoke feelings of stigma and blame in parents. ProAsk is not only intended to accurately calculate risk using a reliable and validated algorithm, but also to reduce the sense that assignment of risk status was a personal judgement by the health visitor. This study showed that this strategy did not fully deliver this intended benefit.

Choosing not to focus on a particular area of behaviour change could also have supported a non-confrontational approach, where no particular parenting practice is construed as contributing to the infant’s higher risk of overweight. But theory and empirical research suggests that goal setting is an important strategy for health behaviour change [46], and that goal attainment is supported by focussing on a single goal, whereas multiple goals may inhibit progress [47]. The results of this study clearly demonstrated a need for specialist training and ongoing staff development and support for practitioners to communicate effectively with parents about risk of child overweight.

The health visiting service varies across the UK, depending on local area provisions. In this study HVs were recruited from two contrasting geographical locations in England, covering both rural and inner city areas. The sample of parents provided balance to the dataset with regard to the infant overweight risk variable. The study sites included areas of high deprivation, and postcode analysis showed the interview sample included families living in the most income deprived and the least income deprived areas in England, as classified by the Income Deprivation Affecting Children Index (IDACI) [48]. However, as reported for the feasibility study, there was a predominance of White British parents in the interview sample, and this is a limitation. One reason for this bias was that language was a significant barrier to recruitment. Since the prevalence of childhood obesity is higher in non-white populations [49] and there are ethnic differences in the modifiable risk factors for childhood obesity [50] future studies will need to include resources for interpreting and translation services.

## Conclusions

MHealth interventions have the potential to engage parents in discussions about childhood obesity prevention. However, health visitors found that using ProAsk and tailoring discussions with parents about their infant’s risk of overweight was challenging. In order to personalise discussions about prevention of childhood obesity the effects of the cognitive biases that undermine effective risk communication need to be minimised. By exposing these barriers, and seeking to understand them from parent and health visitor perspectives, this study takes an important step towards this goal. Future work is needed to translate our understanding of these barriers into strategies that support effective communication between health visitors and parents regarding an infant’s risk of becoming an overweight child.

## Additional files

Additional file 1: Completed consolidated criteria for reporting qualitative studies (COREQ) checklist (DOCX 19 kb)

Additional file 2: **Table S1.** Health Visitor interview schedule. **Table S2.** Parent interview schedule (DOCX 16 kb)

## Abbreviations

HV: Health visitor (public health nurse)
ProAsk: Proactive Assessment of Obesity Risk during Infancy

## References

[1] Estrada E, Eneli I, Hampl S, Mietus-Snyder M, Mirza N, Rhodes E, Sweeney B, Tinajero-Deck L, Woolford SJ, Pont SJ (2014). Children’s hospital association consensus statements for comorbidities of childhood obesity. Child Obes.

[2] Knai C, Lobstein T, Darmon N, Rutter H, McKee M (2012). Socioeconomic patterning of childhood overweight status in Europe. Int J Environ Res Public Health.

[3] Pizzi MA, Vroman K (2013). Childhood obesity: effects on Children's participation, mental health, and psychosocial development. Occup Ther Health Care.

[4] Williams EP, Mesidor M, Winters K, Dubbert PM, Wyatt SB (2015). Overweight and obesity: prevalence, consequences, and causes of a growing public health problem. Curr Obes Rep.

[5] UNICEF WHO World Bank Group (2016). Levels and trends in child malnutrition, 2016.

[6] Commission on Ending Childhood ObesityReport of the Commission on Ending Childhood Obesity. Geneva: World Health Organization; 2016. http://www.who.int/end-childhood-obesity/en/.

[7] Redsell SA, Edmonds B, Swift JA, Siriwardena AN, Weng S, Nathan D, Glazebrook C (2016). Systematic review of randomised controlled trials of interventions that aim to reduce the risk, either directly or indirectly, of overweight and obesity in infancy and early childhood. Matern Child Nutr.

[8] Blake-Lamb TL, Locks LM, Perkins ME, Woo Baidal JA, Cheng ER, Taveras EM (2016). Interventions for childhood obesity in the first 1,000 days a systematic review. Am J Prev Med.

[9] Rosenstock IM (1974). The health belief model and preventive health behaviour.

[10] Redsell SA, Weng S, Swift JA, Nathan D, Glazebrook C (2016). Validation, Optimal Threshold Determination, and clinical utility of the infant risk of overweight checklist for early prevention of child overweight. Child Obes.

[11] Weng SF, Redsell SA, Nathan D, Swift JA, Yang M, Glazebrook C (2013). Estimating overweight risk in childhood from predictors during infancy. Pediatrics.

[12] Han PKJ, Piccirillo J, Gutheil C, Williams D, Wartak MM, Dufault C, Hallen S, Lucas FL, Joekes K (2016). Development and evaluation of an online risk communication teaching program for medical students. Med Sci Educ.

[13] French DP, Cameron E, Benton JS, Deaton C, Harvie M (2017). Can communicating personalised disease risk promote healthy behaviour change? A systematic review of systematic reviews. Ann Behav Med.

[14] Chi MTH, Wylie R (2014). The ICAP framework: linking cognitive engagement to active learning outcomes. Educ Psychol.

[15] Free C, Phillips G, Galli L, Watson L, Felix L, Edwards P, Patel V, Haines A (2013). The effectiveness of Mobile-health technology-based health behaviour change or disease management interventions for health care consumers: a systematic review. PLoS Med.

[16] Raaff C (2015). Dietitians’ perceptions of communicating with preadolescent, overweight children in the consultation setting: the potential for e-resources. J Hum Nutr Diet.

[17] Raaff C, Glazebrook C, Wharrad H (2014). A systematic review of interactive multimedia interventions to promote children's communication with health professionals: implications for communicating with overweight children. BMC Med Inform Decis Mak.

[18] Manuguerra M, Petocz P (2011). Promoting student engagement by integrating new technology into tertiary education: the role of the iPad. Asian Soc Sci.

[19] Sung E, Mayer RE (2013). Online multimedia learning with mobile devices and desktop computers: an experimental test of Clark’s methods-not-media hypothesis. Comput Human Behav.

[20] Thomson R, Edwards A, Grey J (2005). Risk communication in the clinical consultation. Clin Med (Lond).

[21] Ferguson M, Brandreth M, Brassington W, Leighton P, Wharrad H (2016). A randomized controlled trial to evaluate the benefits of a multimedia educational program for first-time hearing aid users. Ear Hear.

[22] Manning JC, Latif A, Cooper J, Horsley A, Armstrong M, Wharrad H (2015). Our care through our eyes’: a mixed-methods, evaluative study of a service-user, co-produced education programme to improve inpatient care of children and young people admitted following self-harm. BMJ Open.

[23] Manning JC, Carter T, Latif A, Horsley A, Cooper J, Armstrong M, Crew J, Wood D, Callaghan P, Wharrad H (2017). Our care through our eyes’. Impact of a co-produced digital educational programme on nurses’ knowledge, confidence and attitudes in providing care for children and young people who have self-harmed: a mixed-methods study in the UK. BMJ Open.

[24] Glazebrook C, Garrud P, Avery A, Coupland C, Williams H (2006). Impact of a multimedia intervention “Skinsafe” on patients’ knowledge and protective behaviors. Prev Med.

[25] Redsell SA, Rose J, Ablewhite J, Swift JA, Siriwardena ANS, Nathan D, Weng S, Wharrad H, Atkinson P, Watson V (2017). Digital technology to facilitate proactive assessment of obesity risk during infancy (ProAsk): a feasibility study. BMJ Open.

[26] Parker I (1998). Social Constructionism, Discourse and Realism.

[27] Tong A, Sainsbury P, Craig J (2007). Consolidated criteria for reporting qualitative research (COREQ): a 32-item checklist for interviews and focus groups. Int J Qual Health Care.

[28] Cetateanu A, Jones A (2014). Understanding the relationship between food environments, deprivation and childhood overweight and obesity: evidence from a cross sectional England-wide study. Health Place.

[29] NHS Digital (2016). National Child Measurement Programme: England 2015/16 school year.

[30] Miller WR, Rollnick S (2013). Motivational interviewing : helping people change.

[31] Bowen DJ, Kreuter M, Spring B, Cofta-Woerpel L, Linnan L, Weiner D, Bakken S, Kaplan CP, Squiers L, Fabrizio C (2009). How we design feasibility studies. Am J Prev Med.

[32] Braun V, Clarke V (2006). Using thematic analysis in psychology. Qual Res Psychol.

[33] Boyatzis RE (1998). Transforming qualitative information: thematic analysis and code development.

[34] Fade SA, Swift JA (2011). Qualitative research in nutrition and dietetics: data analysis issues. J Hum Nutr Diet.

[35] Gagnon MP, Ngangue P, Payne-Gagnon J, Desmartis M (2016). M-health adoption by healthcare professionals: a systematic review. J Am Med Inform Assoc.

[36] Riva S, Camerini A-L, Allam A, Schulz PJ (2014). Interactive sections of an internet-based intervention increase empowerment of chronic Back pain patients: randomized controlled trial. J Med Internet Res.

[37] R. Fields (2011). The top 10 challenges facing healthcare workers.

[38] Chen S, Chaiken S, Chaiken S, Trope Y (1999). The heuristic-systematic model in its broader context. Dual process theories in social psychology.

[39] Lord C, Ross L, Lepper M (1979). Biased assimilation and attitude polarization: the effects of prior theories on subsequently considered evidence. J Pers Soc Psychol.

[40] Tversky A, Kahneman D (1974). Judgment under uncertainty: heuristics and biases. Science.

[41] Usher-Smith J, Emery J, Hamilton W, Griffin SJ, Walter FM (2015). Risk prediction tools for cancer in primary care. Br J Cancer.

[42] Wang C, Gordon ES, Norkunas T, Wawak L, Liu CT, Winter M, Kasper RS, Christman MF, Green RC, Bowen DJ (2016). A randomized trial examining the impact of communicating genetic and lifestyle risks for obesity. Obesity.

[43] Redsell SA, Atkinson PJ, Nathan D, Siriwardena AN, Swift JA, Glazebrook C (2011). Preventing childhood obesity during infancy in UK primary care: a mixed-methods study of HCPs’ knowledge, beliefs and practice. BMC Fam Pract.

[44] Bentley F, Swift J, Cook R, Redsell S (2017). “I would rather be told than not know” - a qualitative study of parents views on identifying infants at future risk of childhood overweight and obesity. BMC Public Health.

[45] Wolfson JA, Gollust SE, Niederdeppe J, Barry CL (2015). The role of parents in public views of strategies to address childhood obesity in the United States. Milbank Q.

[46] Mann T, de Ridder D, Fujita K (2013). Self-regulation of health behavior: social psychological approaches to goal setting and goal striving. Health Psychol.

[47] Sheeran P (2016). The intention–behavior gap. Soc Personal Psychol Compass.

[48] Department for Communities and Local Government (2015). Official Statistics: English indices of deprivation.

[49] Caprio S, Daniels SR, Drewnowski A, Kaufman FR, Palinkas LA, Rosenbloom AL, Schwimmer JB (2008). Influence of race, ethnicity, and culture on childhood obesity: implications for prevention and treatment: a consensus statement of shaping America's health and the Obesity Society. Diabetes Care.

[50] Woo Baidal JA, Locks LM, Cheng ER, Blake-Lamb TL, Perkins ME, Taveras EM (2016). Risk factors for childhood obesity in the first 1,000 days: a systematic review. Am J Prev Med.

