# Supplementary material for: Proactive Assessment of Obesity Risk during Infancy (ProAsk): a qualitative study of parents’ and professionals’ perspectives on an mHealth intervention
Source: BMC Public Health. 2019 Mar 12;19:294. doi: 10.1186/s12889-019-6616-5 (PMC6417230; doi:10.1186/s12889-019-6616-5)
Supplement: Supplementary file 1 — Completed consolidated criteria for reporting qualitative studies (COREQ) checklist (DOCX 19 kb) [file 12889_2019_6616_MOESM1_ESM.docx]

**Additional File 1: Completed consolidated criteria for reporting qualitative studies (COREQ) checklist**

| **Item number** | **Guide questions/description** |  |
| --- | --- | --- |
| **Domain 1: Research team and reflexivity**  **Personal Characteristics** | | |
| 1.Interviewer/facilitator | Which author/s conducted the interview or focus group? | JR, JA |
| 2. Credentials | What were the researcher’s credentials? | JR: BSc(Hons), PhD  JA: BSc(Hons), PhD |
| 3. Occupation | What was their occupation at the time of the study? | JR: Research Assistant; JA: Research Fellow |
| 4. Gender | Was the researcher male or female? | Female |
| 5. Experience and training | What experience or training did the researcher have? | The researchers both held doctorates and had training and experience of conducting and analysing qualitative research interviews. |
| **Relationship with participants** | |  |
| 6. Relationship established | Was a relationship established prior to study commencement? | Over the 6 months of the feasibility study the researchers intermittently met the health visitors, supporting them to identify potential participants and deliver ProAsk to parents who had consented to take part in the study. The researchers visited all of the parent participants in their homes to brief them about the ProAsk study before they consented to take part, and collected baseline data. They had no further contact until they invited parents to take part in the interviews when their infants were 6 months old. |
| 7. Participant knowledge of the interviewer | What did the participants know about the researcher? | The participants knew the researchers’ roles and the organisations for whom they worked, and had met the researcher on a previous occasion. |
| 8. Interviewer characteristics | What characteristics were reported about the interviewer/facilitator? | Participants reported that the researchers were approachable and friendly. |
| **Domain 2: study design**  **Theoretical framework** | | |
| 9. Methodological orientation and theory | What methodological orientation was stated to underpin the study? | This study was conducted from an interpretative perspective, situated within a critical realist framework. |
| **Participant selection** |  |  |
| 10. Sampling | How were participants selected? | We selected a maximum variation sample. All health visitors who took part in the ProAsk Feasibility study were invited to take part in the interviews. For the parent interviews we used purposive sampling to identify a sample that included parents from all four study sites, wo received their ProAsk assessment from different eight different HVs’, who received an above healthy overweight risk result for their infant (N=6), who received a population overweigh risk result for their infant (N=6). |
| 11. Method of approach | How were participants approached? | Health visitors who had taken part in the ProAsk feasibility sample were emailed or telephoned and invited to take part in the interviews. Parents who had taken part in the feasibility study were telephoned when their infants were six months old and invited to take part in the interviews |
| 12. Sample size | How many participants were in the study? | 12 parents and 15 health visitors took part. |
| 13. Non-participation | How many people refused to participate or dropped out? Reasons? | Seven HVs who had taken part in the feasibility study did not take part in the qualitative interviews. They had left post (N=4), were on leave (N=2), or did not have time to take part in the interviews (N=1). All parents contacted agreed to take part in the interviews. |
| **Setting** |  |  |
| 14. Setting of data collection | Where was the data collected? | Interviews were conducted with parents over the telephone. Parents were in their own homes, researchers were in a quiet office. Interviews with health visitors face to face in a quiet room in the clinic/practice where they worked, or over the telephone in the same setting. |
| 15. Presence of non-participants | Was anyone else present besides the participants and researchers? | No |
| 16. Description of sample | What are the important characteristics of the sample? | All participants were female. Health visitors worked in two geographical areas in England, one predominantly rural, one predominantly urban.  The educational status of parent participants was relatively high, and the infants were predominantly white British. Sixty percent of the mothers and 40% of the fathers were overweight or obese before they had their baby. |
| **Data collection** |  |  |
| 17. Interview guide | Were questions, prompts, guides provided by the authors? Was it pilot tested? | Interview topic guides were developed by FM, SR and JS to cover the areas of focus of a feasibility study[31]. This study reports analysis of data from questions about the acceptability of the intervention. Data concerning feasibility of study methods are reported elsewhere [25]. The interview guides were not piloted because the interviews were developed to be delivered post intervention, however they were reviewed by the full research team, which included a range of clinicians including health visitors. |
| 18. Repeat interviews | Were repeat interviews carried out? If yes, how many? | No repeat interviews were carried out |
| 19. Audio/visual recording | Did the research use audio or visual recording to collect the data? | The interviews were recorded on digital audio recorders |
| 20. Field notes | Were field notes made during and/or after the interview or focus group? | Field notes were made immediately following each interview. |
| 21. Duration | What was the duration of the interviews or focus group? | 20 - 90 minutes. |
| 22. Data saturation | Was data saturation discussed? | Yes |
| 23. Transcripts returned | Were transcripts returned to participants for comment and/or correction? | No. |
| **Domain 3: analysis and findings**  **Data analysis** | | |
| 24. Number of data coders | How many data coders coded the data? | Two, but the codes and a selection of the interviews were developed and discussed by five team members (JS, CG, JR, SR, ANS). |
| 25. Description of the coding tree | Did authors provide a description of the coding tree? | Yes. |
| 26. Derivation of themes | Were themes identified in advance or derived from the data? | Themes were derived from the data using an inductive, interpretive approach |
| 27. Software | What software, if applicable, was used to manage the data? | QSR NVivo 10 |
| 28. Participant checking | Did participants provide feedback on the findings? | Consistent with the researchers’ interpretative, relativist approach, the researcher’s analytical interpretations were not returned to participants for ‘member checking’. However, the researchers did provide feedback to participants on the findings of the study, in the form of written summaries, presentations and discussions. |
| **Reporting** |  |  |
| 29. Quotations presented | Were participant quotations presented to illustrate the themes / findings? Was each quotation identified? | Yes, in boxes for each theme. Each quotation is identified with a pseudonym, |
| 30. Data and findings consistent | Was there consistency between the data presented and the findings? | Yes |
| 31. Clarity of major themes | Were major themes clearly presented in the findings? | Yes |
| 32. Clarity of minor themes | Is there a description of diverse cases or discussion of minor themes? | Yes – diverse cases. |
