# Supplementary material for: Proactive Assessment of Obesity Risk during Infancy (ProAsk): a qualitative study of parents’ and professionals’ perspectives on an mHealth intervention
Source: BMC Public Health. 2019 Mar 12;19:294. doi: 10.1186/s12889-019-6616-5 (PMC6417230; doi:10.1186/s12889-019-6616-5)
Supplement: Supplementary file 2 — Table S1. Health Visitor interview schedule. Table S2. Parent interview schedule (DOCX 16 kb) [file 12889_2019_6616_MOESM2_ESM.docx]

**Additional file 2**

**Supplementary Table 1: Health Visitor interview schedule**

|  |
| --- |
| Intro and welcome |
| RA: Thank you for your time during the study, and for talking to me today. Now we have finished the first trial, we are looking to see how we might be able to improve it, and how Health Visitors like you felt about the whole process. We really are interested in your experience of this, so please don’t worry about making suggestions or telling us if anything didn't work well for you. I would like to start by asking a bit about your experience of approaching parents to participate in the ProAsk trial: |
| Please would you start by describing your experience of approaching parents and carers to participate in the ProAsk trial? |
| What did you find challenging? |
| Did that surprise you? |
| How did you manage that? |
| Would you give me an example? |
| What did you find straightforward? |
| Did that surprise you? |
| Would you give me an example? |
| Were there any parents who you didn’t approach? |
| Can you tell me why you felt unable to approach them? |
| Did you have sufficient time to talk to parents about the study during the home visit? |
| At what point did you bring it up? |
| Why did you choose that time? |
| How did you find explaining the nature of the study? |
| Did you have any concerns about the sensitive nature of the issue of bodyweight? |
| How did parents and carers respond to you introducing the study and asking for their participation? |
| Would you give me an example of a positive response? |
| Would you give me an example of a negative response? |
| How did you manage negative responses? |
| Overall, did you feel confident about approaching parents and carers? |
| Why do think that is? |
| Did you feel you had enough training before being asked to approach parents and carers? |
| Did you feel you had enough support to do this? |
| What would have been more useful? |
| I’d like to move onto discuss your motivations for helping us with this research. |
| Why did you agree to participate in the ProAsk trial? |
| What do you think about the targeting of infants at risk of childhood obesity? |
| What do you believe are the potential benefits? |
| What do you believe are the potential problems? |
| Taking you back to the beginning of the year, you attended a training prior to the start of the project. During this training, you covered information on delivering the intervention and also on motivational interviewing. |
| Have you used elements of the training during the study? |
| What parts of the training have you used? |
| Have you revisited any elements of the training materials? |
| Which? |
| How did you find the training in terms of the |
| Length? |
| Content? |
| How could we make the training more useful to the intervention? |
| If you participated in any post-training supervision sessions, please tell us about those: |
| I’d like to discuss your experience of delivering the ProAsk intervention. Firstly, how was the experience of identifying children? |
| Was that difficult/easy? |
| Why do think that was? |
| Was that a common issue you faced? |
| Did you have any different experiences? |
| How did families react? |
| Why do you think that was? |
| Did you have any different experiences? |
| Did reaction vary according to the child’s risk status? |
| Did you find yourself adapting the language or process with any families? |
| Why do you think you did that? |
| Do you think there was anything that could have helped you identify children? |
| Secondly, how was the experience of using the therapeutic wheel with families? |
| Was that difficult/easy? |
| Why do think that was? |
| Was that a common issue you faced? |
| Did you have any different experiences? |
| How did families react? |
| Why do you think that was? |
| Did you have any different experiences? |
| Did reaction vary according to the child’s risk status? |
| Did you find yourself adapting the language or process with any families? |
| Why do you think you did that? |
| Do you think there was anything that could have helped you work with families? |
| Do you think the advice on the wheel was effective? |
| Why is that? |
| What would you suggest? |
| Have you changed the way you practice since being involved in the ProAsk study? |
| Can you give me an example of how your practice has changed? |
| Information about future projects |
| As you know this project was run as a feasibility trial and we hope to run a full-scale trial in the future. Looking forward, |
| Do you think that there were any unintended consequences for yourself of being involved in ProAsk? |
| Do you think that there were any unintended consequences for the families of being involved in ProAsk? |
| How appropriate is the way in which recruitment for the ProAsk intervention has been designed? |
| Do you believe that it is appropriate for HV to approach parents and carers in the future? |
| Why is that? |
| How appropriate is the design of the ProAsk intervention? |
| Do you think the process of identification used is appropriate? |
| Do you think the therapeutic wheel used is appropriate? |
| What would you suggest? |
| We are also interested in thinking about how well the ProAsk approach might fit into a HV’s normal activities. |
| Do you think that is something that HV would find useful? |
| Why is that? |
| Do you think that it is something that could be integrated into a HV’s current role? |
| What might be needed to facilitate this? |
|  |

**Supplementary Table 2: Parent interview schedule**

| *NB: This interview schedule is an overview of the kinds of questions that may be asked during the follow up interviews with the families. The overall goal is to find out the experience of the approach that we took during the study and consider additional factors that the research team may not have considered. These interviews will last approximately half an hour, and be conducted by a research assistant attached to the project. Each area will be covered broadly, and some of the prompts under each section will be used if appropriate for that particular individual.* |
| --- |
| Intro and welcome |
| RA: Thank you for your time during the study, and for talking to me today. Now we have finished the first trial, we are looking to see how we might be able to improve it, and how parents like you felt about the whole process. We really are interested in your experience of this, so please don’t worry about making suggestions or telling us if anything didn't work well for you. I would like to start by asking a bit about your experience of being recruited to the ProAsk trial |
| RA: How did you feel about being asked to participate? |
| Allow parent to speak freely, however, if the conversation slows, and they have not touched on the following subject, use the following prompts. |
| Prompts |
| Did you feel that you had sufficient information to make a decision? |
| How did you feel about being approached by a HV during the 6-8 week visit? |
| What did you understand was the purpose of the study? |
| Why did you decide to participate? |
| Did you have any concerns at this stage? |
| Is there anything that would have improved your experience of being recruited to the study |
| Can we move on to your experience of using the ProAsk tool with your health visitor? How did you feel about the ProAsk Interview with the Health Visitor? |
| Prompts |
| What did you think about the questions that the HV asked you about risk factors for child overweight and obesity? |
| What do you think about the language we use in the programme? Would you prefer us to use words like obesity risk or risk of being an unhealthy weight or anything else? |
| How did you feel about the feedback of your baby’s risk? |
| What did you understand about what this feedback meant for your child? |
| Was there anything that could have improved the way this feedback was given? |
| During the ProAsk interview, what topics do you remember covering with the HV? |
| Prompts |
| How did you feel about these topics? |
| What about the therapeutic wheel that you saw on the tablet? |
| Was there any thing that made the suggestions difficult to follow? |
| What could have made the advice easier to follow? |
| Did your interaction with the HV change your behaviour in any way? |
| Prompts |
| What kinds of things have you done differently? |
| How has it affected your confidence as parent? |
| Who, if anyone, did you talk to about your involvement in the ProAsk trial, your baby’s identification and the kinds of strategies you talked about with your health visitor? |
| Who? |
| Why? |
| What did they say? |
| Information about future projects |
| Please ask all questions in this section |
| As you know this project was run as a feasibility study to see whether it would be practical and acceptable for the team to carry out a larger randomised trial of ProAsk. This would mean asking all parents of young babies in the practices involved in the study to agree to using the ProAsk tool to identify their baby’s risk of overweight. Parents of infants with risk factors for child overweight would be then be allocated to either receiving the usual HV care plus ProAsk intervention or just HV care as usual. We would then follow up the mothers and babies for a year to see if the ProAsk interview and information helped parents of infants with risk factors for childhood overweight to manage their weight gain |
| With your experience of being involved in the feasibility study how would you would you feel about being approached to take part in a future trial? |
| Would you recommend a friend to take part in a future study? |
| Do you think you would agree to be randomised (equal chance of receiving the ProAsk interview and advice or usual care)? |
| If we have a parents’ advisory panel for the next study would you be happy to be approached to take part. We would ask the panel to review the information materials and questionnaires for the larger study. |
| Thank you for your time during the study, and today in talking with us about your experiences. |
